# Correction: Sundararajan, V., et al. SNAI1-Driven Sequential EMT Changes Attributed by Selective Chromatin Enrichment of RAD21 and GRHL2. *Cancers* 2020, *12*, 1140

**DOI:** 10.3390/cancers12123777

**Published:** 2020-12-15

**Authors:** Vignesh Sundararajan, Ming Tan, Tuan Zea Tan, Qing You Pang, Jieru Ye, Vin Yee Chung, Ruby Yun-Ju Huang

**Affiliations:** 1 Center for Translational Medicine, Cancer Science Institute of Singapore, National University of Singapore, Singapore 117599, Singapore; csivsun@nus.edu.sg (V.S.); tmluanbu1989@163.com (M.T.); csittz@nus.edu.sg (T.Z.T.); pqy@u.nus.edu (Q.Y.P.); vinyeechung@gmail.com (V.Y.C.); 2 Department of Obstetrics and Gynaecology, Yong Loo Lin School of Medicine, National University of Singapore, Singapore 119077, Singapore; 3 School of Medicine & Graduate Institute of Oncology, College of Medicine, National Taiwan University, Taipei 10051, Taiwan; jieruye@ntu.edu.tw

The authors wish to make the following corrections to this paper [1]:

Section 2.1, paragraph 2, line 12, (Figure 1G) should be (Figure 1F,G); (Figure 1H) should be (Figure 1F,H).

Section 2.2, paragraph 2, first sentence: “Our results show that the expression of ERBB3 was significantly downregulated in *SNAI1*-OVCA420 cells at the mRNA and protein levels” should be “Our results show that the expression of ERBB3 was significantly downregulated in *SNAI1*-OVCA420 cells at the mRNA and protein levels (Figure 2B,D)”.

Section 2.2, paragraph 2, line 3, (Figure 3B,D) should be (Figure 2B,D); line 5, (Figure 3C,D) should be (Figure 2C,D); line 7, (Figure 3E,F) should be (Figure 2E,F).

Section 2.3, paragraph 1, line 21, (Figure 3D) should be (Figure 2D).

Section 2.4, paragraph 1, “we tested whether GRHL2 would play a role in altering the RAD21-mediated enrichment along the *PERP* and *ERBB3* loci in our EMT spectrum model” should be “we tested whether GRHL2 would play a role in altering the RAD21-mediated enrichment along the *PERP* and *ERBB3* loci in our EMT spectrum model (Figure 4A)”.

Section 2.4, paragraph 1, last sentence: “with a modest reduction of *PERP* levels (Figure 4A).” should be “with a modest reduction of *PERP* levels (Figure 4B,D)”.

Section 2.4, paragraph 2, first sentence: “a modest increase *PERP* transcript levels (Figure 4A)” should be “a modest increase *PERP* transcript levels (Figure 4C)”.

Section 2.4, paragraph 2, third sentence: “the intron1 (P12) regions in GRHL2 knockdown cells (Figure 4B, left)” should be “the intron1 (P12) regions in GRHL2 knockdown cells (Figure 4E, left)”.

Section 2.4, paragraph 2, fourth sentence: “the intron1 (E20) amplicon regions along the *ERBB3* loci (Figure 4B, right)” should be “the intron1 (E20) amplicon regions along the *ERBB3* loci (Figure 4E, right)”.

Section 2.4, paragraph 2, fifth sentence: “the 3′UTR/poly-A-tail (P18) amplicon regions along the PERP loci (Figure 4C, left)” should be “the 3′UTR/poly-A-tail (P18) amplicon regions along the *PERP* loci (Figure 4F, left)”; “the intron1 (E19 and 20) amplicon regions along the *ERBB3* loci (Figure 4C, right)” should be “the intron1 (E19 and 20) amplicon regions along the *ERBB3* loci (Figure 4F, right)”.

Section 3, paragraph 1, last sentence, “whereas expressions of TWIST1 and ZEB1/2 are higher in cell-line IM and M phenotypes” should be “whereas expressions of TWIST1 and ZEB1/2 are higher in cell-line IM and M phenotypes [8]”.

The authors would like to apologize for any inconvenience caused to the readers by these changes.
