# Supplementary material for: SNAI1-Driven Sequential EMT Changes Attributed by Selective Chromatin Enrichment of RAD21 and GRHL2
Source: Cancers (Basel). 2020 May 2;12(5):1140. doi: 10.3390/cancers12051140 (PMC7281482; doi:10.3390/cancers12051140)
Supplement: Supplementary file 1 [file cancers-12-01140-s001.pdf]

Supplementary Material: SNAI1-Driven Sequential EMT Changes Attributed by Selective Chromatin Enrichment of RAD21 and GRHL2

Vignesh Sundararajan, Ming Tan, Tuan Zea Tan, Qing You Pang, Jieru Ye, Vin Yee Chung and Ruby Yun-Ju Huang

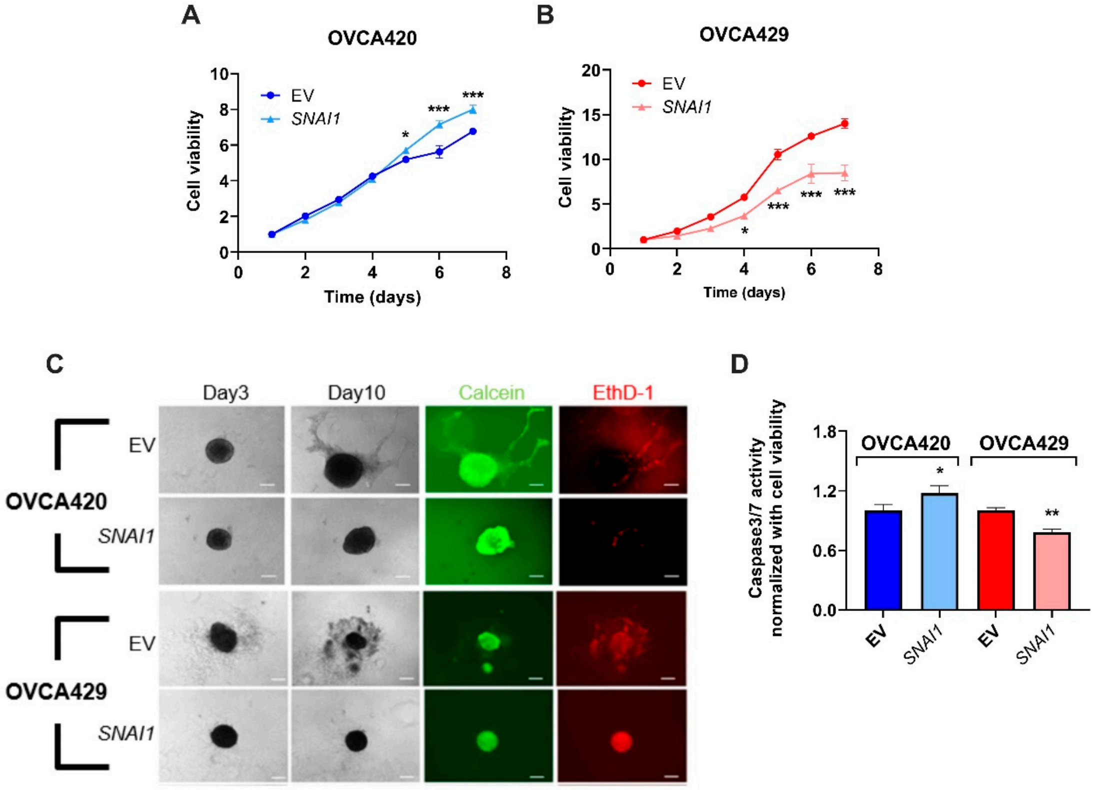

Figure S1. *SNAI1* overexpressing cells in ovarian cancer cells induce differential functional changes. (**A**,**B**) Line graphs representing cell viability (*y*-axis) over time (*x*-axis) of control (EV) and *SNAI1* overexpression clones, that are normalized with time point Day 1. (**C**) Phase contrast images of control (EV) and *SNAI1* overexpression clones grown as spheroids with the addition of spheroid formation agent for three days in round bottom ULA culture plates (Day 3) and subsequently embedded in invasion matrigel until Day 10. Green channels indicate the Calcein-AM (Calcein) signals for live cells while red channels indicate the ethidium homodimer-1 (EthD-1) for dead cells. Scale bars represent 100 µm. (**D**) Control (EV) and *SNAI1* overexpression clones were seeded at a density of 10,000 cells per well in flat bottom ULA culture plates for 3 wells per clone. After 72 h, cell viability for live cells was first measured by Luminescent Cell Viability Assay followed by the measuring the caspase 3/7 activity for dying cells. Caspase 3/7 activity was divided by cell viability for the same well. Y-axis stands for the normalized caspase 3/7 activities for *SNAI1* overexpression cells compared to their respective controls. Statistical significance was determined by unpaired *t*-tests; * $p < 0.05$; ** $p < 0.01$.

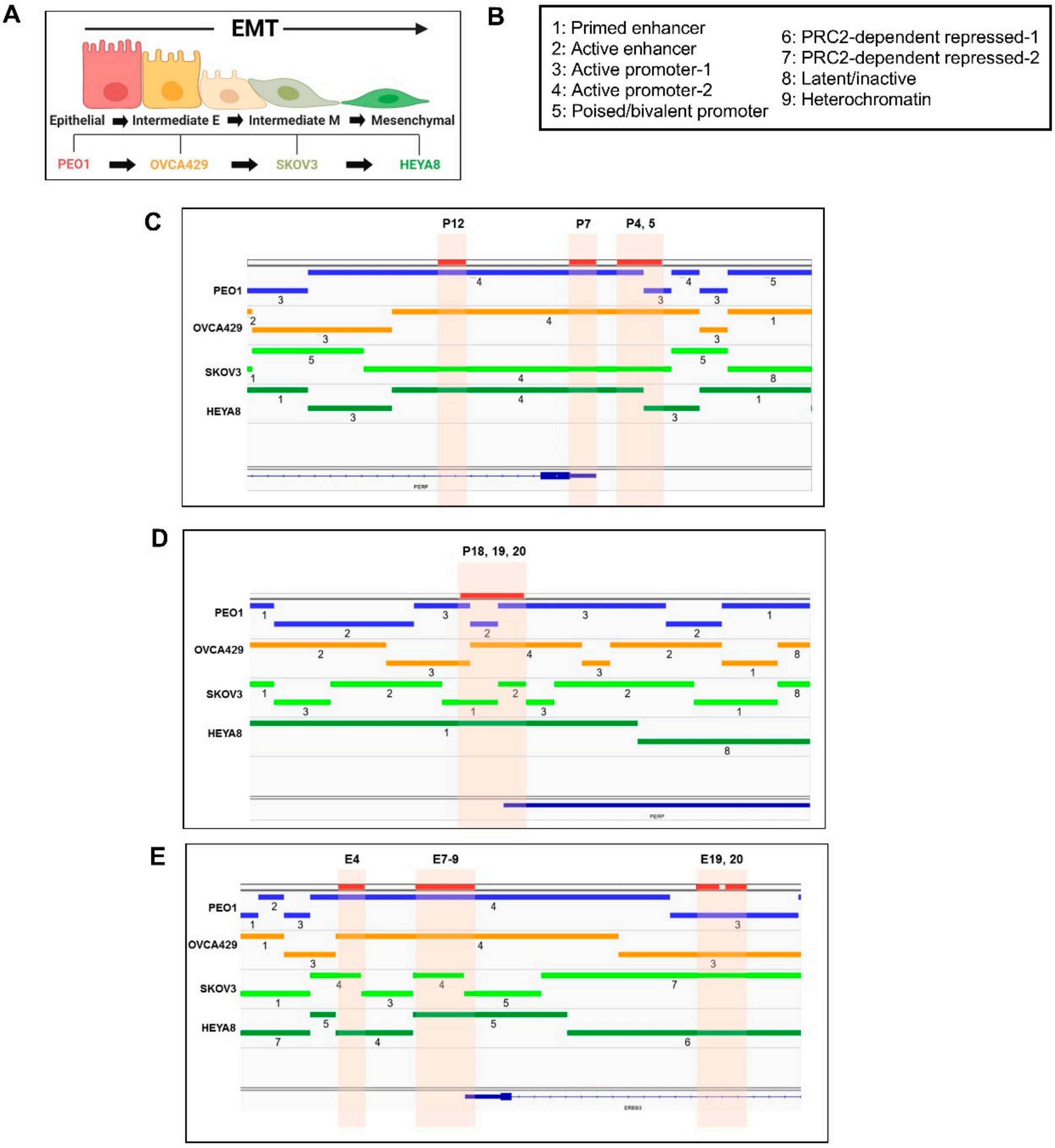

Figure S2. Chromatin states at *PERP* and *ERBB3* amplicon regions in four-cell-line EMT spectrum model. (**A**) Illustration showing the four-cell line model representing cellular states along the EMT spectrum. PEO1 (Epithelial), OVCA429 (Intermediate epithelial), SKOV3 (Intermediate mesenchymal), and HEYA8 (Mesenchymal). (**B**) Nine ChromHMM states encompassing combinatorial histone H3 chromatin immunoprecipitation (ChIP)-sequencing that were previously described by our group [1]. (**C**–**E**) Snapshots of human *PERP* (**B**,**C**) and *ERBB3* (**D**) loci showing ChromHMM state (indicated in number codes depicted in Figure S5A) in the four-cell-line EMT spectrum model: Amplicon regions were highlighted in red.

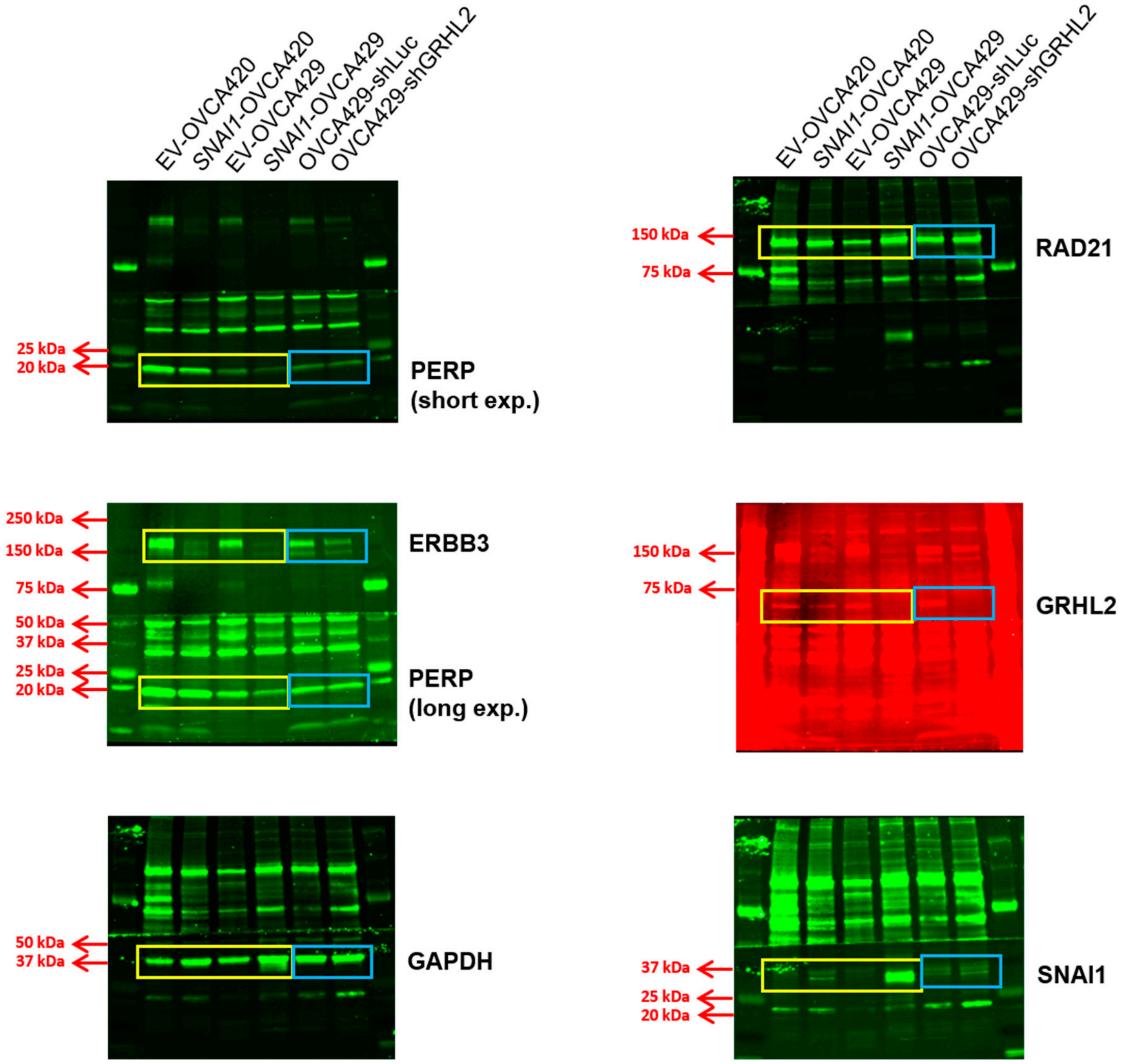

Figure S3. Full-length blots. Bands that are indicated in yellow used to construct Figure 2D and those indicated in blue are used to construct Figure 4D.

Table S1. List of primers used for ChIP analysis.

ChIP amplicons	Coordinates based on hg 38 human genomic annotation		Primers		Amplicon size	Amplicon position in bps relative to TSS	Genomic location
	Start	End	Forward	Reverse			
P4	138107799	138107992	CAGGAAAGAACAGCATGCAA	CAGCAGTGTGAGATGGGTGT	194	−275 to −469	Promoter
P5	138107673	138107852	AGGAGTGCAGCCTTTATCCA	AGGCGCGTGTTTTGTCTTC	180	−149 to −329	Promoter
P7	138107330	138107524	GCTCTGAGTCACCGGAATCT	CAGCGGATCATGTTGACG	195	−1 to +194	5′UTR
P12	138106390	138106592	CCGGTCAGGCTAGTTCTTGT	ATGCTGGGAAAATGGCATAC	203	+931 to +1134	Intron1
P18	138088442	138088648	GGTGTGGAAAAAGCCAAGAA	CAGGATTCCATTTCTTCACCA	207	+18875 to +19082	3′UTR

P19	138088330	138088513	GGAAACTCAGAAGTGTGTGAGC	CCTTGAGTCTTTGCACTGGA	184	+19010 to +19194	Poly-A tail
P20	138088194	138088381	TGGTTGTGTAATGGGGTGAC	CCACATGGAACTTGCTTTCA	188	+19142 to +19330	Poly-A tail
E4	56079034	56079243	AAGGGATTTGAAATGCAAGG	GACGCGGAGAGGACACTAGA	210	−991 to −781	Promoter
E7	56079635	56079809	GCTGGGTGGATGAATTATGG	CGCCCATTAACCAAATCACT	175	−390 to −215	Promoter
E8	56079780	56079946	AGGGGAGTTGAGTGATTTGGT	GATGGGGCTCACCCTAATTT	167	−245 to −78	5′UTR
E9	56079891	56080099	GAATCTCGACCTCCCCTTG	GAGAGAGAGGGAGGGAGGAA	209	−134 to +75	5′UTR
E19	56081820	56081997	GGCTGGAGATTCTGGCTCTA	AAGGGAGCACAAACAACACC	178	+1795 to +1973	Intron1
E20	56082044	56082211	CTCAGGGTAGCAGGGAACTG	CGTACACATTGGTCCTGCTG	168	+2019 to +2187	Intron1

Table S2. List of primers used for qPCR analysis.

Gene	Catalog no.	Ref Seq Accession no.
ACTB	PPH00073E	NM_001101.3
B2M	PPH01094E	NM_004048.2
GAPDH	PPH00150E	NM_002046.3
HPRT1	PPH01018B	NM_000194.2
RPL13A	PPH01020B	NM_012423.2
GRHL2	PPH18929F	NM_024915.3
SNAI1	PPH02459B	NM_005985
SNAI2	PPH02475A	NM_003068
TWIST1	PPH02132A	NM_000474.3
ZEB1	PPH01922A	NM_030751.5
ZEB2	PPH09021B	NM_014795
CDH1	PPH00135E	NM_004360.3
ERBB3	PPH00463B	NM_001982.3
PERP	PPH00596F	NM_022121.5

All primers except RAD21 was purchased from Qiagen. RAD21 primer sequences were obtained from a previous report [2], forward primer (TGACTTTGATCAGCCACTGC) and reverse primer (TCTCACGATCATCCATTCCA).

Table S3. List of antibodies used in this study.

Antibody	Vendor	Cat. No#	Application
BHLHE40	Novus Biologicals	NB100-1800	ChIP
E-cadherin	BD Transduction Laboratories	610182	Immunofluorescence, Western blotting
ERBB3	Abcam	ab20161	Western blotting
GAPDH	Sigma-Aldrich	G9545	Western blotting
GATA3	Cell Signaling Technology	5852	ChIP
GRHL2	Sigma-Aldrich	HPA002820	ChIP, western blotting
HDAC2	Abcam	ab51832	ChIP
HNF4α	Abcam	ab41898	ChIP
IgG (Rabit)	Cell Signaling Technology	2729	ChIP
IgG (Mouse)	Santa Cruz	sc-2025	ChIP

NR2F2	Perseus	PP-H7147-00	ChIP
PERP	Abcam	ab5986	Western blotting
RAD21	Abcam	ab992	ChIP, western blotting
SNAI1	Cell Signaling Technology	3879	Western blotting
TWIST1	Santa Cruz	sc-81417	Western blotting
Vimentin	Dako	M7020	Western blotting
ZEB1	Cell Signaling Technology	3396	Western blotting
ZEB2	Santa Cruz	sc-48789	Western blotting

Supplementary Materials and Methods

Cell Proliferation Assay

Cells were seeded in 96-well plates at 1000 cells per well. At indicated time points, proliferation rate was measured using MTS reagent mix (#G5430, Promega, Madison, WI, USA), and absorbance recorded at 490 nm using a plate reader (Tecan infinite 200).

3D Invasion Assay

3D invasion assay was performed using 96-well 3D Spheroid BME Cell Invasion Assay from Cultrex, Gaithersburg, MD, USA (#3500-096-K) following manufacturer's protocol. Briefly, cells were mixed with kit supplied ECM and seeded into ultra-low attachment plates. The plates were then centrifuged at 500 g for 5 minutes and incubated at 37 °C for 72 hours for spheroid formation. After that, kit supplied BME matrix was added and pre-incubated on ice for 10 minutes, followed by centrifugation at 500 g, 4 °C for 5 minutes. The plates were then cultured for 7 days at 37 °C. Subsequently, cells were stained for 1 hour with Calcein AM (#C3100MP, Life Technologies, California, CA, USA) and Ethidium Homodimer-1 (#E1169, Life Technologies) and imaged using Zeiss Axio Imager M2 fluorescence microscope.

Anoikis Assay (Caspase 3/7 activity)

For the caspase 3/7 activity assay, 10,000 cells/well were seeded in ULA 96-well plates (#7007; Corning). After 72h, 20 µL of CellTiter-Fluor reagent (for cell viability, #TB371; Promega (Madison, WI, USA)) was added to all the wells and the fluorescence was measured after 1h incubation at 37 °C. Subsequently, 100 µL of caspase-Glo 3/7 reagent (#TB323; Promega) was then added and luminescence was measured after 1h incubation at room temperature. The caspase 3/7 activities were divided by the cell viabilities and then normalized to their respective controls.

References

1. Chung, V.Y.; Tan, T.Z.; Ye, J.; Huang, R.Y.-J.; Lai, H.-C.; Kappei, D.; Wollmann, H.; Guccione, E.; Huang, R.Y.-J. The role of GRHL2 and epigenetic remodeling in epithelial-mesenchymal plasticity in ovarian cancer cells. *Commun. Boil.* **2019**, *2*, 272–15, doi:10.1038/s42003-019-0506-3.
2. Yun, J.; Song, S.; Kim, H.; Han, S.; Yi, E.C.; Kim, T. Dynamic cohesin-mediated chromatin architecture controls epithelial–mesenchymal plasticity in cancer. *EMBO Rep.* **2016**, *17*, 1343–1359, doi:10.15252/embr.201541852
